# Normal Skin Cells Increase Aggressiveness of Cutaneous Melanoma by Promoting Epithelial-to-Mesenchymal Transition via Nodal and Wnt Activity

**DOI:** 10.3390/ijms222111719

**Published:** 2021-10-29

**Authors:** Gustavo Untiveros, Lindsay Dezi, Megan Gillette, Julia Sidor, Luigi Strizzi

**Affiliations:** 1 Department of Pathology, College of Graduate Studies, Midwestern University, Downers Grove, IL 60515, USA; guntiv@midwestern.edu; 2 Department of Biomedical Sciences, College of Graduate Studies, Midwestern University, Downers Grove, IL 60515, USA; ldezi11@midwestern.edu; 3 Chicago College of Osteopathic Medicine, Midwestern University, Downers Grove, IL 60515, USA; mgillette53@midwestern.edu (M.G.); jsidor12@midwestern.edu (J.S.); 4 Department of Pathology, College of Graduate Studies, Chicago College of Osteopathic Medicine, Midwestern University, Downers Grove, IL 60515, USA

**Keywords:** melanoma, normal skin cells, co-culture, epithelial-to-mesenchymal transition, Nodal, Wnt

## Abstract

Melanoma is a lethal form of skin cancer triggered by genetic and environmental factors. Excision of early-stage, poorly aggressive melanoma often leads to a successful outcome; however, left undiagnosed these lesions can progress to metastatic disease. This research investigates whether the exposure of poorly aggressive melanoma to certain normal skin cells can explain how non-metastatic melanoma becomes more aggressive while still confined to the skin. To this end, we used a serial co-culture approach to sequentially expose cells from two different, poorly aggressive human melanoma cell lines against normal cells of the skin beginning with normal melanocytes, then epidermal keratinocytes, and finally dermal fibroblasts. Protein extraction of melanoma cells occurred at each step of the co-culture sequence for western blot (WB) analysis. In addition, morphological and functional changes were assessed to detect differences between the serially co-cultured melanoma cells and non-co-cultured cells. Results show that the co-cultured melanoma cells assumed a more mesenchymal morphology and displayed a significant increase in proliferation and invasiveness compared to control or reference cells. WB analysis of protein from the co-cultured melanoma cells showed increased expression of Snail and decreased levels of E-cadherin suggesting that epithelial-to-mesenchymal transition (EMT) is occurring in these co-cultured cells. Additional WB analysis showed increased levels of Nodal protein and signaling and signs of increased Wnt activity in the co-cultured melanoma cells compared to reference cells. These data suggest that interaction between poorly aggressive melanoma cells with normal cells of the skin may regulate the transition from localized, poorly aggressive melanoma to invasive, metastatic disease via Nodal and/or Wnt induced EMT.

## 1. Introduction

The incidence of melanoma is increasing worldwide, which places this disease and potential therapeutic approaches at the forefront of cancer research [1]. Specifically, cutaneous melanoma is an aggressive and deadly skin cancer that arises from the malignant transformation of cutaneous melanocytes, the pigmented cells that reside along the dermal-epidermal junction of the skin [2]. The pathogenesis of melanoma is multifactorial, involving environmental and genetic factors. The major environmental cause is exposure to ultraviolet (UV) radiation [3]. UV-B radiation from sunlight [4] and UV-A radiation from artificial sources, such as tanning beds [5], induce genetic damage in the skin melanocytes that may progress to melanoma. Regarding genetic factors, fair-skinned, red-haired individuals are more susceptible to damage from UV radiation than those who have dark skin pigmentation due to polymorphisms in the melanocortin 1 receptor (MC1R) gene, which lead to reduced melanin production [6,7]. Molecular abnormalities can drive melanoma growth by stimulating proliferation, clonal survival and synthesis of growth factors and other molecules that ultimately modify the normal, microenvironment of the skin and how cells in this microenvironment communicate with each other [8,9].

The Clark Model has been used to describe distinct steps that occur during the progression of melanoma. This progression initiates with abnormal proliferation of melanocytes that lead to the formation of a dysplastic nevus. The degree of atypia in dysplastic nevi can become severe enough that it transitions into the early stage, in situ or radial growth phase (RGP) melanoma [10]. RGP melanoma commonly shows loss of function mutations in tumor suppressor genes and cell cycle regulator genes like CCND1, which further perpetuates melanoma growth [10]. Mutations in KIT, BRAF, NRAS and cyclin-dependent kinase inhibitor 2A (CDKN2A) also participate in melanoma progression [11] as do mutations that lead to excessive PI3K-Akt signaling [12]. In fact, many of these deregulated signaling pathways represent novel therapeutic targets [13]. Progress in immunology has identified aberrant expression of immune check point regulators, such as programmed death-1 (PD-1) and cytotoxic T-lymphocyte associated protein 4 (CTLA-4), that can dampen host anti-tumor immune response and facilitate melanoma growth. This has led to the development of immune checkpoint inhibitors and improved cancer immunotherapy for melanoma treatment [14].

Eventually, as the melanoma cells begin to cross the basement membrane and invade the deeper layers of skin, they enter what is referred to as the vertical growth phase (VGP). During VGP, melanoma cells can gain access to blood vessels and lymphatics and spread to distant sites of the body, giving rise to advanced stage or metastatic melanoma [10]. In addition, the Breslow score or stage is commonly used to evaluate the transition from RGP to VGP by measuring the thickness of melanoma [15]. As this classification system highlights, only a few millimeters separate early-stage, treatable melanoma from aggressive, intractable invasive disease.

A long-held view for melanoma tumor progression encompasses a one-way model of gene expression changes. Such a model, however, fails to explain the molecular signaling heterogeneity that characterizes melanoma [16]. This can be largely the result of various signaling cues and crosstalk from the surrounding microenvironment that can lead to changes in growth rate and rapid increase in aggressiveness [9,17]. The identification of the sources of these specific cues within the tumor microenvironment and, most importantly, the timing of their expression during the different stages of melanoma progression remains unclear. It is crucial, therefore, to identify the pro-aggressive stimulus (or stimuli) that switches melanoma from a local, poorly aggressive state to an invasive form and determine when, during this progression, these factors come into play. This can aid the design of approaches that would improve the effectiveness of melanoma treatment before it becomes an intractable metastatic disease.

One of the mechanisms involved during cancer cell invasion is epithelial-to-mesenchymal transition (EMT) [18,19]. During this process, cancer cells become less adhesive and assume a more mesenchymal-like morphology, which facilitates their capacity to invade, migrate to different microenvironments in the body, and form metastasis [19]. The molecular mechanisms that regulate EMT are diverse and context specific. For example, in embryonic development, EMT plays an important role during gastrulation and organ development [20] and is regulated by different growth factors and embryonic morphogens including Nodal, a member of the transforming growth factor signaling pathway beta (TGF-β) family of proteins [21]. Moreover, Nodal expression in melanoma has been associated with increased plasticity and aggressiveness [22] and shown to regulate melanoma cell migration in an EMT-like process via ERK1/2 signaling [23].

Regulation of cellular adhesion plays an important role during EMT. For instance, E-cadherins, transmembrane proteins responsible for cell adhesion and intracellular signaling, normally form a complex with β-catenin (β-cat) and suppress tumor cell motility [24,25]. E-cadherins, however, can become downregulated via specific transcription factors like the zinc finger proteins Snail, Slug, and Zeb, which bind to E-boxes in the *CDH1* gene responsible for E-cadherin expression and cause inactivation [26,27,28]. Another regulator known as Twist, which is a member of the basic helix loop helix family of transcription factors, is a key player during mesodermal development capable of inducing the expression of key proteins and cytokines that can facilitate EMT [29,30,31]. These transcription factors are acted upon by different signaling pathways including TGF-beta members, Notch, Hedgehog, and Wnt [32].

Like Nodal, the Wnt signaling pathway is important for normal development and has been shown to be involved in cancer growth and spread [33]. Moreover, Wnt signaling via β-cat is also a major regulatory component of melanocyte differentiation and cell cycle progression and has also been shown to play a role in melanoma progression [34,35]. Loss of E-cadherin causes intracellular accumulation of β-cat and increased Wnt signaling activity. Normally, β-cat levels are kept under control via phosphorylation. Phosphorylated-β-catenin (p-β-cat) is then ubiquitinated and eliminated via proteasome degradation. However, excess non-degraded β-cat can translocate to the nucleus and activate downstream Wnt-dependent transcription factors that promote cell survival, growth, and invasion of different cancer cell types, including melanoma [36,37].

The goal of this research is to investigate how the normal skin microenvironment can affect the aggressiveness of early-stage melanoma. Our results show that poorly aggressive human melanoma cells acquire morphologic, functional, and molecular changes that suggest induction of EMT through increased activity of Nodal and Wnt signaling following exposure to normal skin cells. Thus, cues from the normal skin microenvironment may trigger the transition from early-stage localized melanoma into metastatic disease.

## 2. Results

### 2.1. Morphology and Function Are Affected in Poorly Aggressive Melanoma Cells When Co-Cultured with Normal Skin Cells

**Figure 1:**
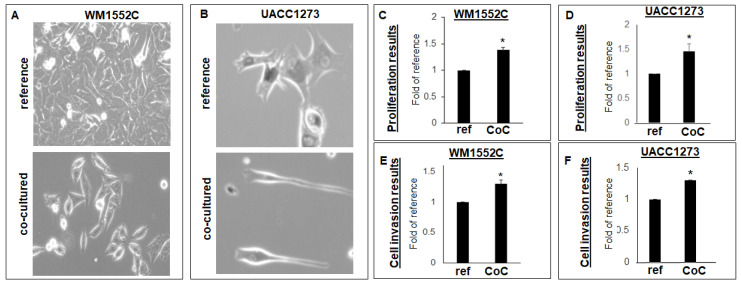
Morphologic and functional changes in poorly aggressive melanoma cells exposed to normal skin cells. Poorly aggressive, human melanoma cell lines WM1552C ((**A**), 10× original magnification) and UACC1273 ((**B**), 20× original magnification) show morphologic change from the native, epithelioid aspect (reference) to mesenchyme-like after co-culture with normal human skin cells (co-cultured). Results from MTT assay show increased metabolic activity in WM1552C (**C**) and UACC1273 (**D**) after co-culture with normal human skin cells compared to reference cells (ref). Results from cell invasion assay show increased invasiveness of WM1552C (**E**) and UACC1273 (**F**) after co-culture with normal human skin cells compared to reference cells (ref) (* *p* < 0.05). Assay results are representative of at least three independent experiments, each performed in quadruplicate.

After subjecting the poorly aggressive melanoma cell lines WM1552C and UACC1273 to a co-culture sequence with epidermal keratinocytes and fibroblasts, both melanoma cell lines acquired a more spindle-like shape appearance compared to the respective, more epithelioid-shaped, control cells (Figure 1A,B). Results from MTT assay show significant increases in viability for both WM1552C (1.39-fold of control +/− 0.05, *p* < 0.05) and UACC1273 (1.46-fold of control +/− 0.16, *p* < 0.05) compared to the respective control cells (Figure 1C,D). Results from cell invasion experiments also show significant increase in cell invasiveness for both WM1552C (1.3-fold of control +/− 0.06, *p* < 0.05) and UACC1273 (1.28-fold of control +/− 0.01, *p* < 0.05) compared to the respective non-cultured control cells (Figure 1E,F). These results suggest that poorly aggressive melanoma cells become more mesenchymal in appearance and show increased metabolic activity when exposed to normal epidermal keratinocytes and dermal fibroblasts.

### 2.2. Molecular Changes Associated with Epithelial-to-Mesenchymal (EMT) Occur in Poorly Aggressive Melanoma Cells When Co-Cultured with Normal Skin Cells

**Figure 2:**
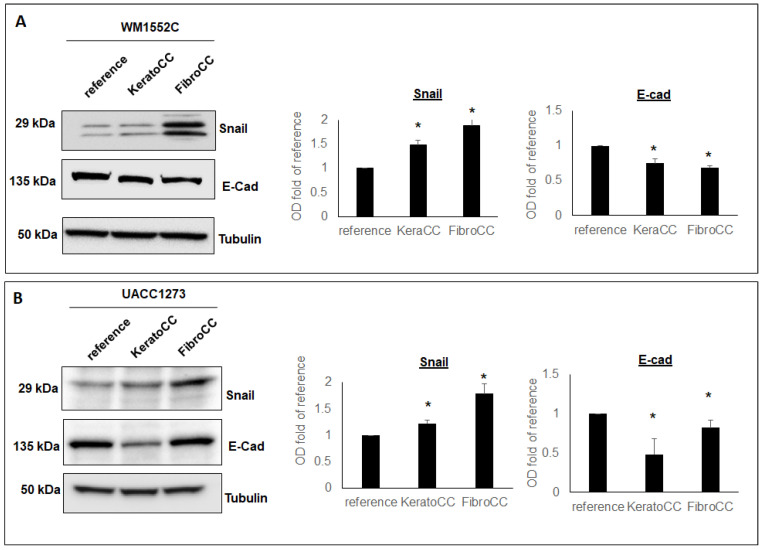
Poorly aggressive melanoma cells acquire molecular changes suggestive of EMT after exposure to normal skin cells. Results from WB and densitometric analysis of WB bands show that the EMT-related transcription factor Snail and the adhesion molecule E-cadherin (E-cad) is decreased in both WM1552C (**A**) and UACC1273 (**B**) poorly aggressive human melanoma cell lines co-cultured with normal human epidermal keratinocytes (KeraCC) and normal dermal fibroblasts (FibroCC) compared to reference cells (* *p* < 0.05). Results are representative of at least two independent experiments, each performed in triplicate.

The morphological changes observed in the co-cultured melanoma cells suggested to us that EMT may be occurring in the poorly aggressive melanoma cells as they are co-cultured with epidermal keratinocytes and dermal fibroblasts. To verify this, we analyzed protein expression of the EMT-related transcription factor Snail and the cellular adhesion molecule E-cadherin in the poorly aggressive melanoma cells co-cultured with epidermal keratinocytes and dermal fibroblasts. Results from WB in Figure 2A show that WM1552C melanoma cells increase Snail expression after exposure to epidermal keratinocytes (1.49-fold of reference +/− 0.085, *p* < 0.05) and dermal fibroblasts (1.88-fold of reference +/− 0.18, *p* < 0.05). These same co-cultured WM1552C cells showed decreased expression in E-cadherin after exposure to epidermal keratinocytes (0.75-fold of reference, +/− 0.07, *p* < 0.05) and dermal fibroblasts (0.69-fold of reference +/− 0.03, *p* < 0.05). In a similar fashion, results in Figure 2B show that UACC1273 melanoma cells increase Snail expression after exposure to epidermal keratinocytes (1.22-fold of reference, +/− 0.06, *p* < 0.05) and dermal fibroblasts (1.79-fold of reference +/− 0.19, *p* < 0.05). Moreover, in UACC1273, E-cadherin expression decreased after exposure to epidermal keratinocytes (0.48-fold of reference, +/− 0.2, *p* < 0.05) and dermal fibroblasts (0.82-fold of reference +/− 0.1, *p* < 0.05). These results show that, after exposure to normal skin cells, poorly aggressive melanoma cells increase expression of the transcription factor Snail, known to negatively regulate cell adhesion. In fact, the same melanoma cells exposed to the normal epidermal keratinocytes and dermal fibroblasts also show decreased expression of the cellular adhesion molecule E-cadherin. Thus, these results suggest that molecular changes associated with the induction of EMT may play an important role during the progression of poorly aggressive melanoma cells as they become exposed to normal cells of the skin.

### 2.3. Nodal Expression and Signaling Activity Increase in Poorly Aggressive Melanoma Cells When Co-Cultured with Normal Skin Cells

**Figure 3:**
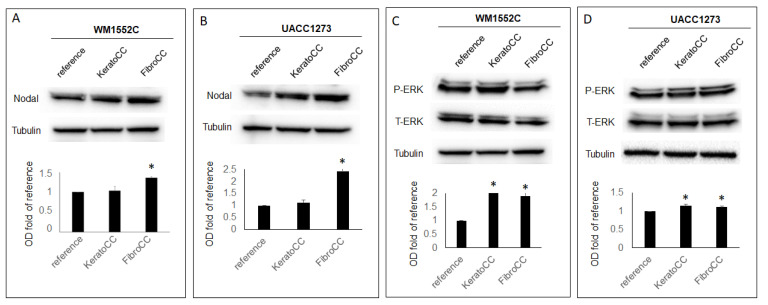
Poorly aggressive melanoma cells show increased expression of Nodal and increased ERK1/2 activity after exposure to normal skin cells. Results from WB and densitometric analysis of WB bands show Nodal expression gradually increasing to significant levels after co-culture of WM1552C (**A**) and UACC1273 (**B**) with normal human epidermal keratinocytes (KeraCC) and normal dermal fibroblasts (FibroCC) compared to reference cells. The same co-culture experiment showed an increase in ERK1/2 phosphorylation in both WM1552C (**C**) and UACC1273 (**D**) (* *p* < 0.05). Results are representative of at least two independent experiments, each performed in triplicate.

Western blot analysis was performed to determine whether the embryonic morphogen Nodal may be implicated in regulating EMT in poorly aggressive melanoma cells exposed to normal skin cells. Our WB results show that both WM1552C and UACC1273 poorly aggressive melanoma cells gradually increased Nodal protein expression during the co-culture sequence with the normal skin cells. In fact, Nodal protein levels significantly increased in WM1552C (1.36-fold of control +/− 0.03, *p* < 0.05) and UACC1273 (2.42-fold of control +/− 0.02, *p* < 0.05) after the final exposure to the dermal fibroblasts (Figure 3A,B). In addition, WM1552C showed significant increases in Erk1/2 signaling activity after co-culture with epidermal keratinocytes (2.1-fold of control +/− 0.02, *p* < 0.05) and dermal fibroblasts (1.9-fold of control +/− 0.2, *p* < 0.05) (Figure 3C). UACC1273 also showed a small but still significant increase in Erk1/2 signaling activity after co-culture with epidermal keratinocytes (1.2-fold of control +/− 0.04, *p* < 0.05) and dermal fibroblasts (1.11-fold of control +/− 0.2, *p* < 0.05) (Figure 3D). These results show that Nodal expression and signaling activity is increased in poorly aggressive melanoma cells exposed to normal epidermal keratinocytes and dermal fibroblasts.

### 2.4. Wnt Signaling Increases in Poorly Aggressive Melanoma When Co-Cultured with Normal Skin Cells

**Figure 4:**
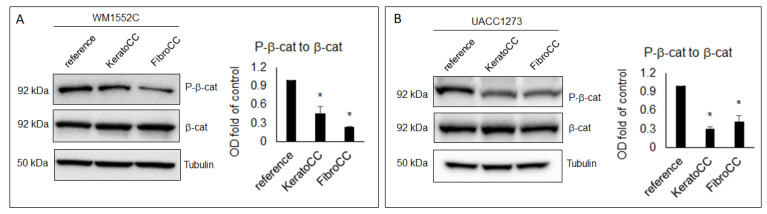
Poorly aggressive melanoma cells show increased Wnt activity after exposure to normal skin cells. Results from WB and densitometric analysis of WB bands show decreases in the ratio of P-β-catenin to total β-catenin (P-β-cat to β-cat), indicating reduced degradation of β-cat, which would lead to increased potential for Wnt signaling in WM1552C (**A**) and UACC1273 (**B**) co-cultured with normal human epidermal keratinocytes (KeraCC) and normal dermal fibroblasts (FibroCC) compared to reference cells (* *p* < 0.05). Results are representative of at least two independent experiments, each performed in triplicate.

To determine whether an alternate pathway could also be involved in regulating EMT in poorly aggressive melanoma cells exposed to normal skin cells, we performed WB analysis to evaluate Wnt signaling by analyzing P-β-cat levels. Our WB results show a significant decrease in P-β-cat levels in WM1552C after co-culture with epidermal keratinocytes (0.45-fold of control +/− 0.12, *p* < 0.05) and dermal fibroblasts (0.23-fold of control +/− 0.02, *p* < 0.05) (Figure 4A). Similarly, UACC1273 also showed a significant decrease in P-β-cat levels after co-culture with epidermal keratinocytes (0.31-fold of control +/− 0.03, *p* < 0.05) and dermal fibroblasts (0.42-fold of control +/− 0.1, *p* < 0.05) (Figure 4B). These results show that P-β-cat levels are reduced and, therefore, Wnt signaling is increased in poorly aggressive melanoma cells exposed to normal epidermal keratinocytes and dermal fibroblasts.

## 3. Discussion

One of the most challenging issues faced in melanoma research is understanding how poorly aggressive RGP melanoma suddenly develops into the aggressive and deadly VGP disease. In fact, the Breslow scoring system, which is used to predict outcome based on melanoma thickness, highlights how melanoma can transition from a localized excisable and curable lesion into advanced metastatic disease with just a few millimeters of increased growth. Clearly, RGP melanoma is receiving signals to transition into a more aggressive disease while still within the confines of the skin microenvironment. In this study, we hypothesized that cues from resident normal cells of the skin can stimulate change in early-stage, poorly aggressive melanoma cells that lead to the increased aggressiveness necessary for disease progression. For this purpose, we used a transwell co-culture system to grow poorly aggressive melanoma cells first in the presence of normal human epidermal keratinocytes and then normal human dermal fibroblasts. This technique allowed exposure of the melanoma cells to any molecular stimuli from the keratinocytes or fibroblasts while at the same time keeping the cells physically separated and allowing us to then analyze and harvest the melanoma cells without any concern regarding cross contamination of cell types. The results from our experiments show that poorly aggressive melanoma cell lines WM1552C and UACC1273 experienced morphological, functional, and molecular changes associated with invasive melanoma when co-cultured with normal epidermal keratinocytes and normal dermal fibroblasts. These observations suggest that the co-cultured melanoma cells had embarked along the path of increased aggressiveness following exposure to the normal skin cells.

We initially found that, after the co-culture sequence, both WM1552C and UACC1273 were no longer epithelioid in shape but had acquired a more spindle-shaped morphology while also becoming more metabolically active and showing increased invasiveness. These changes suggested to us that the melanoma cells were experiencing EMT. EMT is a well-established process whereby cancer cells become more aggressive by assuming a more mesenchyme-like, motile phenotype [20]. In fact, to allow for increased motility during EMT, molecular changes are necessary to decrease cell adhesion. For example, the transcription factor Snail is upregulated during EMT, resulting in increased degradation of adhesion molecules, such as E-Cad [38]. In fact, we observed increased expression of Snail and concomitant decrease in E-cad expression in the co-cultured poorly aggressive melanoma cells. This is further evidence that supports the ability of normal cells of the skin microenvironment, such as keratinocytes and fibroblasts, to induce EMT in poorly aggressive melanoma cells.

Many signaling pathways have been shown to induce EMT in cancer cells, including TGF-β family of proteins. Nodal is an embryonic morphogen and TGF-β family member. Although the primary role for Nodal is to regulate embryonic development and organ specification, it has also been shown to be expressed in several different cancer types [39]. In patient tumor samples, Nodal expression has been shown to be correlated with advanced-stage disease [40,41,42]. In malignant cells, Nodal expression is associated with increased proliferation, plasticity, and invasiveness [43]. Moreover, Nodal has been shown to be capable of playing a role during EMT via activation of ERK1/2 signaling [23]. In our study, we found increased levels of Nodal protein and activated P-ERK1/2 in both poorly aggressive melanoma cell lines after co-culture with the keratinocytes and fibroblasts. This suggests that the EMT-related changes observed in the co-cultured poorly aggressive melanoma cells may be the result of increased Nodal expression and signaling in the melanoma cells exposed to the normal skin cells. To our knowledge, this is the first study to show increased Nodal expression in non-aggressive melanoma cells exposed to normal cells of the skin.

Optimal Nodal function requires binding to its co-receptor Cripto-1 during the regulation of normal embryonic development [44]; however, both Nodal and Cripto-1 have been shown to signal independently of each other [45,46]. Like Nodal, Cripto-1 has also been shown to be associated with progression of different types of human cancers and proposed as a potential therapeutic target [47]. Whether Nodal/Cripto-1 dynamics play a combined role in the same cancer remains unclear. In a seminal study by Postovit et al., where Nodal was described for the first time as a potential regulator of melanoma cell progression, Cripto-1 levels were low or barely detected in the Nodal expressing melanoma cells studied, and the authors concluded that Nodal signaling most likely occurred in a Cripto-1-independent fashion in melanoma [48]. In a different study, Cripto-1 expression in patient samples detected by immunohistochemistry (IHC) failed to show differences in Cripto-1 levels between early and late-stage melanoma [49]. In another study, although Nodal expression detected by IHC was significantly greater in metastatic melanoma versus localized cutaneous melanoma, Cripto-1 levels did not change between the same two groups [50]. Similar results for Nodal and Cripto-1 immunostaining were observed for human oral squamous cell carcinoma (OSCC) [51]. Interestingly, this same study showed that simultaneous inhibition of both Nodal and Cripto-1 resulted in significant reduction of viability, signaling, and invasiveness of OSCC cells compared to treatment with either Nodal or Cripto-1 inhibitor alone. In our present study, not only was Cripto-1 barely detectable by WB, we also could not detect any significant change in Cripto-1 levels in the co-cultured melanoma cells tested (data not shown). Overall, these data suggest that Nodal expression level appears to be more susceptible to microenvironmental changes than its co-receptor Cripto-1 and that perhaps Nodal may require only minimum amounts of Cripto-1 co-expression to carry out pro-tumorigenic effects in melanoma.

It is well known that in malignant disease like melanoma, multiple signaling pathways converge to regulate redundant activities [52]. In fact, this may explain why cancer recurrence is more likely to occur with therapies that target only one pathway compared to multimodality approaches that target several different pathways. For this reason, we explored whether, in addition to Nodal, there could be an alternate signaling pathway that would validate the changes we observed in the poorly aggressive melanoma cells exposed to the skin keratinocytes and fibroblasts. We found that the co-cultured, poorly aggressive melanoma cells showed decreased levels of P-β-cat, which is evidence that Wnt activity was increased in the melanoma cells after they were exposed to the keratinocytes and fibroblasts. Like Nodal, Wnt signaling also plays an important role during development and is expressed in multiple human cancers where it is capable of regulating proliferation and metastatic spread [53]. For example, oral squamous cell carcinoma cell lines with induced, constitutively active Wnt signaling are shown to exhibit EMT characteristics, such as cell morphology changes from polygonal to spindle-shaped, cell adhesion rearrangements, and increased migration and invasion [54]. Additionally, it is well reported that human breast cancer also exhibits upregulation of β-cat, as well as aberrant activation of the Wnt/β-cat signaling pathway. Moreover, data shows that breast cancer cell lines with elevated β-cat levels, due to inappropriate stabilization of β-cat, also display distinct EMT characteristics, such as spindle-shaped cell morphology and an increase in Snail [55]. Besides melanoma, oral squamous cell carcinoma, and breast carcinoma, several other human cancers have been shown to display Wnt-induced EMT by similar molecular mechanism as reported in our results [56]. Investigation of this pathway continues to be a prevalent area of research in hopes to improve therapeutic modalities in the treatment of cancer.

## 4. Materials and Methods

### 4.1. Cell Cultures

Two poorly aggressive human melanoma cell lines, WM1552C (purchased from Rockland Immunochemicals, Limerick, PA, USA) and UACC1273 (a generous gift from Dr. Richard Seftor, University of West Virginia, WV, USA) were used for this study. Cells were maintained in RPMI1640 media (GenClone, San Diego, CA, USA) supplemented with 5% FBS (Seradigm, Batavia, IL, USA). Epidermal melanocytes (ATCC, Manassas, VA, USA, PCS-200-013), keratinocytes (ATCC, PCS-200-010), and dermal fibroblasts (ATCC, PCS-201-012) were grown each according to vendor specifications. All cells were incubated in 37 °C and 5% CO_2_ conditions.

### 4.2. Serial Co-Culture

To mimic the exposure of poorly aggressive melanoma cells to different normal cells of the skin, we used a standard Transwell cell co-culture system (Corning, Corning, NY, USA). For this, approximately 150,000 WM1552C or UACC1273 melanoma cells were seeded in the insert of the 6-well plate transwell system, which is lined at the bottom with a porous membrane. This setup allows for the poorly aggressive melanoma cells seeded in the topside insert to be serially transferred to different fixed bottom wells containing the different normal skin cell types (Supplementary Figure S1A). The proximity between the transferable top insert and the fixed bottom well allows for molecular crosstalk between the poorly aggressive melanoma cells and specific normal cell type at each co-culture step without the cells ever coming into direct, physical contact. Thus, WM1552C or UACC1273 cells were co-cultured for 24 h at each step of the co-culture sequence beginning with normal melanocytes, followed by keratinocytes and then dermal fibroblasts. At each step of the co-culture sequence, portions of the transwell inserts were collected and analyzed for microscopic morphology, proliferation, invasion, and protein expression (Supplementary Figure S1B). The poorly aggressive melanoma cells were initially co-cultured with normal melanocytes to simulate the clinical scenario where early-stage, poorly aggressive melanoma cells are surrounded by normal melanocytes while still localized in the skin. Therefore, these cells were used as our control or reference cells.

### 4.3. MTT Assay

To evaluate the effect of serial co-culture on the proliferation of the poorly aggressive melanoma cells, a standard 4,5-dimethylthiazol-2-yl-2,5-diphenyltetrazolium bromide (MTT) assay kit (Promega, Madison, WI, USA) was used following manufacturer instructions. Briefly, cells were incubated at 37 °C for 4 h with MTT dye solution. A solubilization solution/stop mix was added for 1 h incubation at 37 °C. Thus, the intensity of the color of the formazan produced (proportional to the number of metabolically active, proliferating cells) was read using the Beckman Coulter DTX 880 Multimode spectrophotometer to determine optical density (OD) at 570 nm. For this experiment, treatment was performed on 8 replicates per experimental condition and repeated at least three times. The final fold change in cell proliferation was calculated as a ratio of OD of co-cultured cells relative to their respective reference cells.

### 4.4. Cell Invasion/Migration Assay

WM1552C or UACC1273 cells from each co-culture step were seeded in 3.0um pore, 24-well transwell plates coated with Collagen Type 4 (Col IV) (Corning) at 100,000 cells/transwell. Transwell plates were incubated overnight at 37 °C and 5% CO_2_. Media were removed and transwells were transferred to a 24-well plate with 1X PBS. Cotton swabs moistened with PBS were used to gently remove non-migrated residual cells from the upper surface of the transwell. Transwells with the migrated cells on the underside of the Col IV coated membrane were transferred to a 24-well plate with PBS and washed. These transwells were then added to a 24-well plate with 650 µL/well of crystal violet solution and incubated on a rocking platform for 10 min. The transwells were washed 5X with PBS and transferred to a 24-well plate with 400 µL/well of 10% acetic acid solution used to extract the stain. The plate was again placed on a rocker for 10 min at RT in the dark. Finally, 150 µL of extracted stain from each sample was transferred to a 96-well plate and OD was read at 590 nm. The final fold change in cell proliferation was calculated as a ratio of OD of co-cultured cells relative to the respective reference cells.

### 4.5. Protein Extraction and Western Blot

For protein extraction, the co-cultured WM1552C or UACC1273 poorly aggressive melanoma cells were washed 3 times with PBS. Then 20 µL of standard radioimmunoprecipitation assay buffer (RIPA) (Pierce, Waltham, MA, USA) containing protease inhibitors (Pierce) was added to each well containing the co-cultured melanoma cells, which were then scrapped and left to incubate for 10 min on ice. Protein lysates were collected and spun at 16,000× *g* for 30 min at 4 °C. For WB, 30 ug of protein per sample were separated by SDS-PAGE electrophoresis and transferred to PVDF membranes (Millipore, Burlington, MA, USA). Membranes were then washed 3 times in TBST and then blocked with 5% non-fat dry milk or 5% bovine serum albumin for 1 h at room temperature. After blocking, membranes were incubated with appropriate dilutions of primary antibody in blocking buffer overnight at 4 °C. The following antibodies/dilutions were used: mouse anti-Nodal/1:1000 (Santa Cruz, sc-81953, Dallas, TX, USA), rabbit anti-phosphorylated-ERK1/2 (p-ERK1/2)/1:1000 (Cell Signaling, Danvers, MA, USA), rabbit anti-ERK1/2/1:1000 (Cell Signaling), mouse anti-β-cat/1:1000 (Novus Biologicals, Centennial, CO, USA); rabbit anti-p-β-cat/1:1000 (Cell Signaling), mouse anti-Snail/1:1000 (Cell Signaling), rabbit anti-E-cadherin/1:1000 (Cell Signaling), and rabbit anti-⍺-tubulin/1:5000 (Cell Signaling). After washing membranes 3 times for 10 min with TBST, they were incubated with appropriate dilutions of conjugated secondary antibodies at room temperature for 1 h. Secondary antibodies used were as follows: anti-mouse 1:5000 (GE Amersham, Marlborough, MA, USA), anti-rabbit 1:5000 (GE Amersham), and anti-goat 1:5000 (R&D Systems, Minneapolis, MN, USA). After washing membranes again 3 times for 10 min with TBST, they were incubated with ECL (Pierce) for development of bands which were imaged using the Bio-Rad Universal Chemidoc system.

### 4.6. Statistical Analysis

GraphPad statistical software was used to perform t-tests for comparing the mean values [+/−standard error of the mean (SEM)] calculated from a minimum of triplicate results from at least two independent experiments between co-cultured and reference control melanoma cells. Results showing a *p* value of less than 0.05 (*p* < 0.05) was considered statistically significant.

## 5. Conclusions

In this study, we used an in vitro serial co-culture technique to model exposure of poorly aggressive melanoma to normal skin cells to understand whether signals from certain cells of the normal skin microenvironment can induce phenotypic and molecular changes reminiscent of the transition from RGP to aggressive VGP melanoma while the lesion is still confined to the skin during early-stage disease and exposed to cues from surrounding normal cells. We show that poorly aggressive melanoma cells exposed to epidermal keratinocytes and dermal fibroblasts acquire phenotypic and molecular changes suggestive of EMT. Moreover, for the first time, we provide molecular evidence that crosstalk with normal epidermal keratinocytes and dermal fibroblasts may result in increased Nodal activity in poorly aggressive melanoma cells. We also show that Wnt signaling is also upregulated in the co-cultured melanoma cells. Thus, our results suggest that EMT could be an important early event triggered by increased Nodal and/or Wnt activity during early-stage melanoma because of crosstalk with the normal cellular components of the skin and that this then sets the stage for progression to invasive VGP melanoma. Further studies are needed to identify the precise molecular signals from the cutaneous microenvironment that induce increased aggressiveness in early-stage melanoma and facilitate the identification of potential therapeutic targets that can halt melanoma progression.

## Data Availability

All data reported are available per request.

## Supplementary Materials

The following are available online at https://www.mdpi.com/article/10.3390/ijms222111719/s1.
