# Supplementary material for: Normal Skin Cells Increase Aggressiveness of Cutaneous Melanoma by Promoting Epithelial-to-Mesenchymal Transition via Nodal and Wnt Activity
Source: Int J Mol Sci. 2021 Oct 29;22(21):11719. doi: 10.3390/ijms222111719 (PMC8583838; doi:10.3390/ijms222111719)
Supplement: Supplementary file 1 [file ijms-22-11719-s001.zip › ijms-1413614-supplementary.pdf]

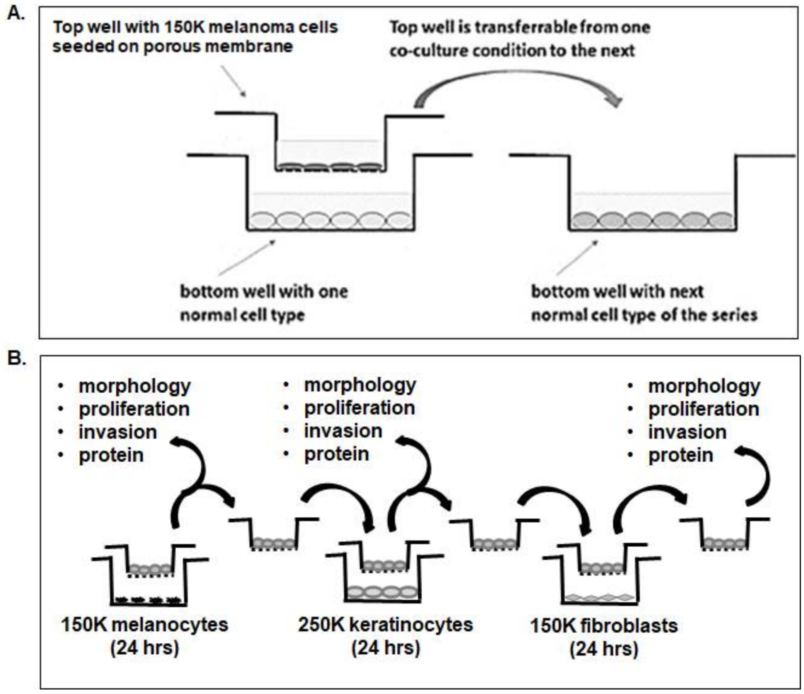

Supplementary Figure S1: (**A**) The poorly aggressive human melanoma cells WM1552C or UACC1273 are co-cultured with normal human melanocytes for 24 hours – this becomes the control or reference cells. (**B**) A portion of the reference cells will continue along the co-culture sequence to be exposed to normal human epidermal keratinocytes for 24 hours followed by exposure to normal human dermal fibroblasts for 24 hours. At each step, cell samples will be analyzed for morphology, proliferation, invasion, and harvested for protein analysis by Western blotting.
